# 2-Amino-5-methyl­pyridinium 1*H*-pyrazole-3,5-dicarboxyl­ate trihydrate

**DOI:** 10.1107/S1600536810041644

**Published:** 2010-10-20

**Authors:** Tara Shahani, Hoong-Kun Fun, Madhukar Hemamalini

**Affiliations:** a X-ray Crystallography Unit, School of Physics, Universiti Sains Malaysia, 11800 USM, Penang, Malaysia

## Abstract

In the title compound, 2C_6_H_9_N_2_^+^·C_5_H_2_N_2_O_4_^2−^·3H_2_O, the 1*H*-pyrazole-3,5-dicarboxyl­ate anion is close to planar [maximum deviation = 0.208 (1) Å]. The two distinct 2-amino-5-methyl­pyridinium cations are also almost planar, with maximum deviations of 0.018 (2) and 0.014 (2) Å. In the crystal, pairs of inter­molecular N—H⋯O and O—H⋯O hydrogen bonds connect neighbouring mol­ecules into dimers, generating *R* ^2^_2_(8) and *R* ^2^_4_(8) ring motifs, respectively. Further inter­molecular N—H⋯O, O—H⋯O and C—H⋯O hydrogen bonds link the mol­ecules into a three-dimensional network.

## Related literature

For background to the chemistry of substituted pyridines, see: Pozharski *et al.* (1997 ▶); Katritzky *et al.* (1996 ▶). For details of hydrogen bonding, see: Jeffrey & Saenger (1991 ▶); Jeffrey (1997 ▶); Scheiner (1997 ▶). For hydrogen-bond motifs, see: Bernstein *et al.* (1995 ▶). For bond-length data, see: Allen *et al.* (1987 ▶). For related structures, see; Xia *et al.* (2007 ▶); King *et al.* (2004 ▶). For details and applications of pyrazole-3,5-dicarb­oxy­lic acid, see: Lee *et al.* (1989 ▶); Chambers *et al.* (1985 ▶); Pan *et al.* (2000 ▶); Pan, Ching *et al.* (2001 ▶); Pan, Frydel *et al.* (2001 ▶).
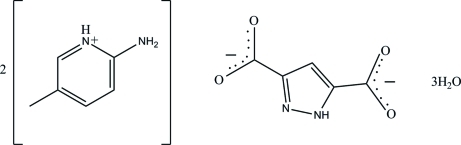


## Experimental

### 

#### Crystal data

2C_6_H_9_N_2_^+^·C_5_H_2_N_2_O_4_^2−^·3H_2_O
*M* *_r_* = 426.44
Triclinic, 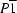

*a* = 7.8985 (1) Å
*b* = 9.2195 (1) Å
*c* = 15.3922 (2) Å
α = 101.942 (1)°
β = 93.883 (1)°
γ = 104.648 (1)°
*V* = 1052.40 (2) Å^3^
*Z* = 2
Mo *K*α radiation
μ = 0.11 mm^−1^
*T* = 100 K
0.47 × 0.24 × 0.21 mm

#### Data collection

Bruker SMART APEXII CCD diffractometer
Absorption correction: multi-scan (*SADABS*; Bruker, 2009 ▶) *T*_min_ = 0.952, *T*_max_ = 0.978
26056 measured reflections
6103 independent reflections
5085 reflections with *I* > 2σ(*I*)
*R*_int_ = 0.026

#### Refinement

*R*[*F* ^2^ > 2σ(*F* ^2^)] = 0.038
*wR*(*F* ^2^) = 0.113
*S* = 1.07
6103 reflections
325 parameters
H atoms treated by a mixture of independent and constrained refinement
Δρ_max_ = 0.44 e Å^−3^
Δρ_min_ = −0.24 e Å^−3^

### 

Data collection: *APEX2* (Bruker, 2009 ▶); cell refinement: *SAINT* (Bruker, 2009 ▶); data reduction: *SAINT*; program(s) used to solve structure: *SHELXTL* (Sheldrick, 2008 ▶); program(s) used to refine structure: *SHELXTL*; molecular graphics: *SHELXTL*; software used to prepare material for publication: *SHELXTL* and *PLATON* (Spek, 2009 ▶).

## Supplementary Material

Crystal structure: contains datablocks global, I. DOI: 10.1107/S1600536810041644/hb5681sup1.cif

Structure factors: contains datablocks I. DOI: 10.1107/S1600536810041644/hb5681Isup2.hkl

Additional supplementary materials:  crystallographic information; 3D view; checkCIF report

## Figures and Tables

**Table 1:** Hydrogen-bond geometry (Å, °)

*D*—H⋯*A*	*D*—H	H⋯*A*	*D*⋯*A*	*D*—H⋯*A*
N2—H1*N*2⋯O4^i^	0.931 (16)	1.871 (16)	2.7912 (12)	169.7 (15)
N4*A*—H3*NA*⋯O1*W*^ii^	0.861 (18)	2.024 (17)	2.8520 (14)	161.2 (17)
N3*B*—H1*NB*⋯O3^iii^	0.900 (17)	1.755 (17)	2.6483 (12)	171.4 (16)
N4*B*—H2*NB*⋯O4^iii^	0.914 (18)	2.022 (18)	2.9323 (13)	173.8 (16)
N4*B*—H3*NB*⋯O3*W*^iv^	0.889 (18)	2.007 (18)	2.8641 (13)	161.6 (17)
N3*A*—H1*NA*⋯O2^iv^	0.942 (18)	1.732 (18)	2.6686 (12)	172.8 (17)
N4*A*—H2*NA*⋯O1^iv^	0.907 (18)	2.106 (18)	3.0021 (13)	169.4 (15)
O1*W*—H1*W*1⋯O3	0.871 (19)	1.902 (19)	2.7517 (12)	164.8 (17)
O1*W*—H2*W*1⋯O3*W*^iv^	0.85 (2)	1.94 (2)	2.7878 (14)	178 (2)
O2*W*—H1*W*2⋯O1	0.850 (18)	2.003 (18)	2.8427 (12)	169.8 (17)
O2*W*—H2*W*2⋯O1^v^	0.858 (18)	1.987 (18)	2.8434 (13)	176.1 (15)
O3*W*—H1*W*3⋯O2	0.888 (17)	1.844 (17)	2.7299 (12)	174.8 (15)
O3*W*—H2*W*3⋯O2*W*^vi^	0.881 (18)	1.900 (18)	2.7758 (13)	172.1 (17)
C10—H10*A*⋯O2*W*	0.93	2.50	3.3986 (15)	164

Symmetry codes: (i) 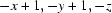; (ii) 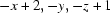; (iii) 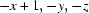; (iv) 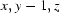; (v) 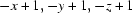; (vi) 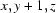.

**Fig. 1.**
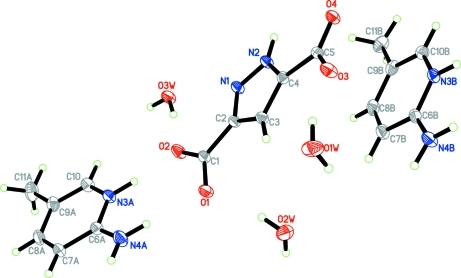
The molecular structure of the title compound, showing 50% probability displacement ellipsoids.

**Fig. 2.**
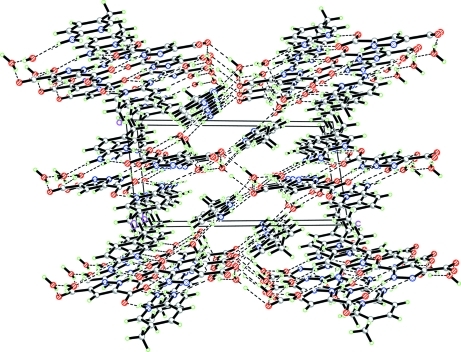
The crystal packing of the title compound, viewed along b axis. Intermolecular hydrogen bonds linked the molecules into three-dimensional network.

## supplementary crystallographic information

### Comment

Pyridine and its derivatives play an important role in heterocyclic chemistry (Pozharski *et al.*, 1997; Katritzky *et al.*, 1996). They are often involved in hydrogen-bond interactions (Jeffrey & Saenger, 1991; Jeffrey, 1997; Scheiner, 1997). Pyrazole-related molecules have attracted considerable attention due to their biological activities (Lee *et al.*, 1989; Chambers *et al.*, 1985). 3,5-Pyrazole dicarboxylic acid (H_2_PzDCA) is a multifunctional ligand; it has multiple coordination sites that allow structures of higher dimensions and it also has abstractable protons that allow various acidity-dependent coordination modes (Pan *et al.*, 2000). A variety of H_2_PzDCA coordination compounds have been synthesized and reported in the literature (Pan, Ching *et al.*, 2001; Pan, Frydel *et al.*, 2001). Since our aim is to study some interesting hydrogen-bonding interactions, the crystal structure of the title compound is presented here.

The asymmetric unit of the title compound, (Fig. 1), consists of two 2-amino-5-methylpyridinium cations, a 1*H*-pyrazole-3-5-dicarboxylate anion and three water molecules. The 1*H*-pyrazole-3,5-dicarboxylate anion and 2-amino-5-methylpyridinium cations are approximately planar with a maximum deviations of 0.208 (1) Å at atom O2 and 0.018 (2) Å at atoms N4A, C11A and 0.014 (2) Å at atom N4B. The torsion angles (O2/C2/C1/N1), (C1–C3/O1), (C3–C5/O3) and (N2/C4/C5/04) are 8.81 (15), 10.46 (16), 4.89 (15) and 4.60 (16)°, respectively. Bond lengths (Allen *et al.*, 1987) and angles are normal and comparable to those related structures (Xia *et al.*, 2007; King *et al.*, 2004).

In the crystal packing (Fig. 2), intermolecular N2—H1N2···O4, N4A—H3NA···O1W, N3B—H1NB···O3, N4B—H2NB···O4, N4B—H3NB···O3W, N3A—H1NA···O2, N4A—H2NA···O1, O1W—H1W1···O3, O1W—H2W1···O3W, O2W—H1W2···O1, O2W—H2W2···O1, O3W—H1W3···O2, O3W—H2W3···O2W and C10—H10A···O2W hydrogen bonds (Table 1) link the molecules into three-dimensional network. Within this network, pairs of intermolecular N3B—H1NB···O3, N4A—H2NA···O1 and O1—H1W2···O2W, O2W—H1W2···O1 hydrogen bonds connect neighbouring molecules to form dimers, generating *R*^2^_2_(8) and *R*^2^_4_(8) (Bernstein *et al.*, 1995) ring motifs, respectively.

### Experimental

A hot methanol/water solution (10/10 ml) of 2-amino-5-methylpyridine (54 mg, Aldrich) and pyrazole-3,5-dicarboxylic acid (78 mg, Merck) were mixed and warmed over a heating magnetic stirrer for a few minutes. The resulting solution was allowed to cool slowly at room temperature and colourless blocks of (I) appeared after a few days.

### Refinement

The hydrogen atoms bound to O atoms were located in a difference map and constrained to ride with their parent atoms, with *U*_iso_(H) = 1.5*U*_iso_(O) [O—H = 0.85 (2)–0.889 (18) Å]. The hydrogen atoms bound to N atoms were located in a difference map and were refined freely [N—H = 0.863 (18)–0.943 (18) Å]. All other H atoms to C were positioned geometrically [range of C—H = 0.93–0.96 Å] with *U*_iso_(H) = 1.2 or 1.5*U*_iso_(C).

### Crystal data

**Table:**

2C_6_H_9_N_2_^+^·C_5_H_2_N_2_O_4_^2^^−^·3H_2_O	*Z* = 2
*M**_r_* = 426.44	*F*(000) = 452
Triclinic, *P*1	*D*_x_ = 1.346 Mg m^−^^3^
Hall symbol: -P 1	Mo *K*α radiation, λ = 0.71073 Å
*a* = 7.8985 (1) Å	Cell parameters from 9892 reflections
*b* = 9.2195 (1) Å	θ = 2.4–35.1°
*c* = 15.3922 (2) Å	µ = 0.11 mm^−^^1^
α = 101.942 (1)°	*T* = 100 K
β = 93.883 (1)°	Block, colourless
γ = 104.648 (1)°	0.47 × 0.24 × 0.21 mm
*V* = 1052.40 (2) Å^3^	

### Data collection

**Table:**

Bruker SMART APEXII CCD diffractometer	6103 independent reflections
Radiation source: fine-focus sealed tube	5085 reflections with *I* > 2σ(*I*)
graphite	*R*_int_ = 0.026
φ and ω scans	θ_max_ = 30.0°, θ_min_ = 2.4°
Absorption correction: multi-scan (*SADABS*; Bruker, 2009)	*h* = −11→11
*T*_min_ = 0.952, *T*_max_ = 0.978	*k* = −12→12
26056 measured reflections	*l* = −21→21

### Refinement

**Table:**

Refinement on *F*^2^	Primary atom site location: structure-invariant direct methods
Least-squares matrix: full	Secondary atom site location: difference Fourier map
*R*[*F*^2^ > 2σ(*F*^2^)] = 0.038	Hydrogen site location: inferred from neighbouring sites
*wR*(*F*^2^) = 0.113	H atoms treated by a mixture of independent and constrained refinement
*S* = 1.07	*w* = 1/[σ^2^(*F*_o_^2^) + (0.0569*P*)^2^ + 0.2775*P*] where *P* = (*F*_o_^2^ + 2*F*_c_^2^)/3
6103 reflections	(Δ/σ)_max_ < 0.001
325 parameters	Δρ_max_ = 0.44 e Å^−^^3^
0 restraints	Δρ_min_ = −0.24 e Å^−^^3^

### Special details

**Table:**

Experimental. The crystal was placed in the cold stream of an Oxford Cyrosystems Cobra open-flow nitrogen cryostat (Cosier & Glazer, 1986) operating at 100.0 (1) K.
Geometry. All e.s.d.'s (except the e.s.d. in the dihedral angle between two l.s. planes) are estimated using the full covariance matrix. The cell e.s.d.'s are taken into account individually in the estimation of e.s.d.'s in distances, angles and torsion angles; correlations between e.s.d.'s in cell parameters are only used when they are defined by crystal symmetry. An approximate (isotropic) treatment of cell e.s.d.'s is used for estimating e.s.d.'s involving l.s. planes.
Refinement. Refinement of *F*^2^ against ALL reflections. The weighted *R*-factor *wR* and goodness of fit *S* are based on *F*^2^, conventional *R*-factors *R* are based on *F*, with *F* set to zero for negative *F*^2^. The threshold expression of *F*^2^ > σ(*F*^2^) is used only for calculating *R*-factors(gt) *etc*. and is not relevant to the choice of reflections for refinement. *R*-factors based on *F*^2^ are statistically about twice as large as those based on *F*, and *R*- factors based on ALL data will be even larger.

### Fractional atomic coordinates and isotropic or equivalent isotropic displacement parameters (Å^2^)

**Table:**

	*x*	*y*	*z*	*U*_iso_*/*U*_eq_	
O1	0.68720 (11)	0.64094 (9)	0.43780 (5)	0.02004 (16)	
O2	0.63845 (11)	0.84955 (9)	0.40067 (5)	0.02212 (17)	
O3	0.70719 (11)	0.24170 (9)	0.10667 (5)	0.02152 (17)	
O4	0.58424 (10)	0.33899 (9)	0.00540 (5)	0.01827 (16)	
N1	0.56503 (12)	0.68973 (10)	0.21937 (6)	0.01756 (18)	
N2	0.56444 (12)	0.58634 (10)	0.14333 (6)	0.01631 (17)	
C1	0.65092 (14)	0.71292 (12)	0.38074 (7)	0.01627 (19)	
C2	0.62433 (13)	0.63055 (11)	0.28462 (6)	0.01510 (19)	
C3	0.66147 (13)	0.49037 (11)	0.24972 (6)	0.01555 (19)	
H3A	0.7032	0.4279	0.2812	0.019*	
C4	0.62226 (13)	0.46589 (11)	0.15823 (6)	0.01420 (18)	
C5	0.63753 (13)	0.33966 (11)	0.08394 (6)	0.01463 (18)	
N3A	0.80196 (12)	0.03401 (11)	0.55694 (6)	0.01923 (18)	
N4A	0.90801 (14)	−0.15177 (12)	0.60538 (7)	0.0248 (2)	
C6A	0.89063 (14)	−0.00860 (13)	0.62071 (7)	0.0192 (2)	
C7A	0.96163 (15)	0.10318 (14)	0.70158 (7)	0.0226 (2)	
H7AA	1.0235	0.0786	0.7475	0.027*	
C8A	0.93848 (15)	0.24717 (14)	0.71159 (8)	0.0239 (2)	
H8AA	0.9838	0.3193	0.7653	0.029*	
C9A	0.84776 (15)	0.29051 (13)	0.64303 (8)	0.0224 (2)	
C10	0.78076 (15)	0.17873 (13)	0.56658 (7)	0.0212 (2)	
H10A	0.7192	0.2017	0.5199	0.025*	
C11A	0.82578 (18)	0.45015 (14)	0.65337 (10)	0.0324 (3)	
H11A	0.7779	0.4614	0.5969	0.049*	
H11B	0.9384	0.5245	0.6729	0.049*	
H11C	0.7470	0.4665	0.6969	0.049*	
N3B	0.26714 (12)	0.00202 (10)	0.01212 (6)	0.01689 (17)	
N4B	0.37262 (14)	−0.08155 (12)	0.13095 (7)	0.02199 (19)	
C6B	0.30472 (14)	0.02005 (12)	0.10101 (7)	0.01719 (19)	
C7B	0.26600 (15)	0.14651 (13)	0.15793 (7)	0.0213 (2)	
H7BA	0.2909	0.1642	0.2197	0.026*	
C8B	0.19158 (15)	0.24211 (12)	0.12087 (8)	0.0214 (2)	
H8BA	0.1650	0.3240	0.1585	0.026*	
C9B	0.15376 (14)	0.22026 (12)	0.02713 (8)	0.0197 (2)	
C10B	0.19410 (14)	0.09771 (12)	−0.02480 (7)	0.0184 (2)	
H10B	0.1712	0.0791	−0.0868	0.022*	
C11B	0.07429 (16)	0.32759 (14)	−0.01255 (9)	0.0264 (2)	
H11D	0.0564	0.2936	−0.0766	0.040*	
H11E	0.1526	0.4299	0.0046	0.040*	
H11F	−0.0369	0.3278	0.0090	0.040*	
O3W	0.52828 (12)	1.04505 (10)	0.31330 (5)	0.02258 (17)	
O2W	0.54335 (13)	0.32242 (10)	0.43022 (6)	0.02698 (19)	
O1W	0.84767 (12)	0.15951 (12)	0.25161 (6)	0.0291 (2)	
H1N2	0.523 (2)	0.6047 (18)	0.0897 (11)	0.027 (4)*	
H3NA	0.968 (2)	−0.176 (2)	0.6462 (12)	0.040 (5)*	
H1NB	0.286 (2)	−0.080 (2)	−0.0250 (11)	0.033 (4)*	
H2NB	0.393 (2)	−0.162 (2)	0.0913 (12)	0.039 (4)*	
H3NB	0.409 (2)	−0.063 (2)	0.1891 (12)	0.038 (4)*	
H1NA	0.749 (2)	−0.037 (2)	0.5028 (12)	0.040 (4)*	
H2NA	0.853 (2)	−0.220 (2)	0.5538 (12)	0.039 (4)*	
H1W1	0.820 (2)	0.200 (2)	0.2082 (13)	0.045 (5)*	
H2W1	0.751 (3)	0.123 (2)	0.2703 (14)	0.053 (6)*	
H1W2	0.591 (2)	0.414 (2)	0.4263 (12)	0.043 (5)*	
H2W2	0.470 (2)	0.330 (2)	0.4684 (13)	0.044 (5)*	
H1W3	0.558 (2)	0.9771 (19)	0.3403 (11)	0.034 (4)*	
H2W3	0.529 (2)	1.128 (2)	0.3540 (12)	0.043 (5)*	

### Atomic displacement parameters (Å^2^)

**Table:**

	*U*^11^	*U*^22^	*U*^33^	*U*^12^	*U*^13^	*U*^23^
O1	0.0295 (4)	0.0177 (4)	0.0135 (3)	0.0084 (3)	0.0007 (3)	0.0032 (3)
O2	0.0349 (4)	0.0168 (4)	0.0150 (3)	0.0118 (3)	−0.0016 (3)	0.0001 (3)
O3	0.0337 (4)	0.0199 (4)	0.0145 (3)	0.0152 (3)	0.0010 (3)	0.0026 (3)
O4	0.0249 (4)	0.0174 (4)	0.0129 (3)	0.0083 (3)	−0.0006 (3)	0.0022 (3)
N1	0.0240 (4)	0.0158 (4)	0.0130 (4)	0.0078 (3)	0.0007 (3)	0.0013 (3)
N2	0.0224 (4)	0.0151 (4)	0.0120 (4)	0.0080 (3)	0.0004 (3)	0.0014 (3)
C1	0.0186 (5)	0.0164 (4)	0.0130 (4)	0.0051 (4)	0.0010 (3)	0.0015 (3)
C2	0.0181 (4)	0.0143 (4)	0.0124 (4)	0.0047 (4)	0.0010 (3)	0.0020 (3)
C3	0.0188 (5)	0.0148 (4)	0.0135 (4)	0.0055 (4)	0.0011 (3)	0.0034 (3)
C4	0.0157 (4)	0.0129 (4)	0.0138 (4)	0.0046 (3)	0.0010 (3)	0.0022 (3)
C5	0.0167 (4)	0.0140 (4)	0.0128 (4)	0.0039 (3)	0.0020 (3)	0.0027 (3)
N3A	0.0224 (4)	0.0195 (4)	0.0143 (4)	0.0057 (3)	−0.0004 (3)	0.0016 (3)
N4A	0.0293 (5)	0.0217 (5)	0.0213 (5)	0.0068 (4)	−0.0069 (4)	0.0037 (4)
C6A	0.0182 (5)	0.0214 (5)	0.0164 (5)	0.0025 (4)	0.0006 (4)	0.0047 (4)
C7A	0.0210 (5)	0.0264 (5)	0.0158 (5)	0.0009 (4)	−0.0030 (4)	0.0037 (4)
C8A	0.0203 (5)	0.0256 (6)	0.0189 (5)	−0.0002 (4)	−0.0002 (4)	−0.0015 (4)
C9A	0.0206 (5)	0.0207 (5)	0.0228 (5)	0.0042 (4)	0.0021 (4)	0.0002 (4)
C10	0.0223 (5)	0.0212 (5)	0.0199 (5)	0.0074 (4)	0.0008 (4)	0.0033 (4)
C11A	0.0317 (6)	0.0207 (6)	0.0391 (7)	0.0074 (5)	−0.0022 (5)	−0.0039 (5)
N3B	0.0194 (4)	0.0146 (4)	0.0160 (4)	0.0062 (3)	0.0015 (3)	0.0005 (3)
N4B	0.0294 (5)	0.0216 (5)	0.0152 (4)	0.0108 (4)	−0.0015 (4)	0.0013 (3)
C6B	0.0168 (4)	0.0158 (5)	0.0168 (5)	0.0028 (4)	0.0016 (3)	0.0012 (4)
C7B	0.0238 (5)	0.0187 (5)	0.0184 (5)	0.0050 (4)	0.0034 (4)	−0.0009 (4)
C8B	0.0209 (5)	0.0152 (5)	0.0260 (5)	0.0047 (4)	0.0065 (4)	−0.0009 (4)
C9B	0.0168 (5)	0.0154 (5)	0.0272 (5)	0.0046 (4)	0.0049 (4)	0.0049 (4)
C10B	0.0184 (5)	0.0174 (5)	0.0194 (5)	0.0048 (4)	0.0021 (4)	0.0048 (4)
C11B	0.0260 (6)	0.0207 (5)	0.0370 (6)	0.0100 (4)	0.0063 (5)	0.0112 (5)
O3W	0.0336 (5)	0.0189 (4)	0.0159 (4)	0.0100 (3)	−0.0003 (3)	0.0032 (3)
O2W	0.0402 (5)	0.0185 (4)	0.0252 (4)	0.0108 (4)	0.0132 (4)	0.0052 (3)
O1W	0.0251 (4)	0.0407 (5)	0.0259 (4)	0.0095 (4)	−0.0003 (3)	0.0182 (4)

### Geometric parameters (Å, °)

**Table:**

O1—C1	1.2637 (12)	C11A—H11A	0.9600
O2—C1	1.2640 (13)	C11A—H11B	0.9600
O3—C5	1.2637 (12)	C11A—H11C	0.9600
O4—C5	1.2511 (12)	N3B—C6B	1.3468 (13)
N1—N2	1.3467 (12)	N3B—C10B	1.3618 (13)
N1—C2	1.3483 (13)	N3B—H1NB	0.897 (17)
N2—C4	1.3572 (12)	N4B—C6B	1.3329 (14)
N2—H1N2	0.929 (16)	N4B—H2NB	0.910 (18)
C1—C2	1.4907 (14)	N4B—H3NB	0.890 (18)
C2—C3	1.4038 (14)	C6B—C7B	1.4193 (14)
C3—C4	1.3798 (13)	C7B—C8B	1.3683 (16)
C3—H3A	0.9300	C7B—H7BA	0.9300
C4—C5	1.4884 (13)	C8B—C9B	1.4153 (16)
N3A—C6A	1.3468 (14)	C8B—H8BA	0.9300
N3A—C10	1.3656 (14)	C9B—C10B	1.3638 (15)
N3A—H1NA	0.943 (18)	C9B—C11B	1.5027 (15)
N4A—C6A	1.3356 (15)	C10B—H10B	0.9300
N4A—H3NA	0.863 (18)	C11B—H11D	0.9600
N4A—H2NA	0.909 (18)	C11B—H11E	0.9600
C6A—C7A	1.4171 (15)	C11B—H11F	0.9600
C7A—C8A	1.3643 (17)	O3W—H1W3	0.889 (18)
C7A—H7AA	0.9300	O3W—H2W3	0.879 (19)
C8A—C9A	1.4155 (17)	O2W—H1W2	0.85 (2)
C8A—H8AA	0.9300	O2W—H2W2	0.86 (2)
C9A—C10	1.3656 (15)	O1W—H1W1	0.87 (2)
C9A—C11A	1.5026 (17)	O1W—H2W1	0.85 (2)
C10—H10A	0.9300		
			
N2—N1—C2	104.08 (8)	N3A—C10—H10A	119.2
N1—N2—C4	112.83 (8)	C9A—C10—H10A	119.2
N1—N2—H1N2	117.6 (9)	C9A—C11A—H11A	109.5
C4—N2—H1N2	129.5 (9)	C9A—C11A—H11B	109.5
O1—C1—O2	123.84 (9)	H11A—C11A—H11B	109.5
O1—C1—C2	117.16 (9)	C9A—C11A—H11C	109.5
O2—C1—C2	119.00 (9)	H11A—C11A—H11C	109.5
N1—C2—C3	111.76 (9)	H11B—C11A—H11C	109.5
N1—C2—C1	121.73 (9)	C6B—N3B—C10B	123.39 (9)
C3—C2—C1	126.47 (9)	C6B—N3B—H1NB	118.8 (11)
C4—C3—C2	104.59 (9)	C10B—N3B—H1NB	117.7 (11)
C4—C3—H3A	127.7	C6B—N4B—H2NB	119.7 (11)
C2—C3—H3A	127.7	C6B—N4B—H3NB	118.8 (11)
N2—C4—C3	106.73 (9)	H2NB—N4B—H3NB	121.1 (16)
N2—C4—C5	122.30 (9)	N4B—C6B—N3B	119.07 (10)
C3—C4—C5	130.96 (9)	N4B—C6B—C7B	123.59 (10)
O4—C5—O3	125.34 (9)	N3B—C6B—C7B	117.33 (10)
O4—C5—C4	118.90 (9)	C8B—C7B—C6B	119.29 (10)
O3—C5—C4	115.76 (9)	C8B—C7B—H7BA	120.4
C6A—N3A—C10	123.07 (10)	C6B—C7B—H7BA	120.4
C6A—N3A—H1NA	120.5 (11)	C7B—C8B—C9B	122.07 (10)
C10—N3A—H1NA	116.5 (11)	C7B—C8B—H8BA	119.0
C6A—N4A—H3NA	118.1 (12)	C9B—C8B—H8BA	119.0
C6A—N4A—H2NA	119.0 (11)	C10B—C9B—C8B	116.49 (10)
H3NA—N4A—H2NA	122.8 (16)	C10B—C9B—C11B	122.09 (10)
N4A—C6A—N3A	119.34 (10)	C8B—C9B—C11B	121.41 (10)
N4A—C6A—C7A	123.26 (10)	N3B—C10B—C9B	121.42 (10)
N3A—C6A—C7A	117.41 (10)	N3B—C10B—H10B	119.3
C8A—C7A—C6A	119.48 (10)	C9B—C10B—H10B	119.3
C8A—C7A—H7AA	120.3	C9B—C11B—H11D	109.5
C6A—C7A—H7AA	120.3	C9B—C11B—H11E	109.5
C7A—C8A—C9A	122.21 (10)	H11D—C11B—H11E	109.5
C7A—C8A—H8AA	118.9	C9B—C11B—H11F	109.5
C9A—C8A—H8AA	118.9	H11D—C11B—H11F	109.5
C10—C9A—C8A	116.20 (10)	H11E—C11B—H11F	109.5
C10—C9A—C11A	121.63 (11)	H1W3—O3W—H2W3	109.1 (15)
C8A—C9A—C11A	122.17 (11)	H1W2—O2W—H2W2	105.7 (17)
N3A—C10—C9A	121.62 (10)	H1W1—O1W—H2W1	105.7 (18)
			
C2—N1—N2—C4	−0.55 (11)	N4A—C6A—C7A—C8A	179.69 (11)
N2—N1—C2—C3	0.16 (11)	N3A—C6A—C7A—C8A	−0.19 (16)
N2—N1—C2—C1	177.98 (9)	C6A—C7A—C8A—C9A	−1.06 (17)
O1—C1—C2—N1	172.05 (10)	C7A—C8A—C9A—C10	1.43 (17)
O2—C1—C2—N1	−8.81 (15)	C7A—C8A—C9A—C11A	−178.63 (11)
O1—C1—C2—C3	−10.46 (16)	C6A—N3A—C10—C9A	−0.66 (17)
O2—C1—C2—C3	168.68 (10)	C8A—C9A—C10—N3A	−0.58 (16)
N1—C2—C3—C4	0.25 (12)	C11A—C9A—C10—N3A	179.48 (11)
C1—C2—C3—C4	−177.44 (10)	C10B—N3B—C6B—N4B	178.55 (10)
N1—N2—C4—C3	0.72 (12)	C10B—N3B—C6B—C7B	−0.34 (15)
N1—N2—C4—C5	−178.32 (9)	N4B—C6B—C7B—C8B	−178.10 (11)
C2—C3—C4—N2	−0.56 (11)	N3B—C6B—C7B—C8B	0.74 (15)
C2—C3—C4—C5	178.37 (10)	C6B—C7B—C8B—C9B	−0.84 (17)
N2—C4—C5—O4	−4.89 (15)	C7B—C8B—C9B—C10B	0.50 (16)
C3—C4—C5—O4	176.33 (10)	C7B—C8B—C9B—C11B	−179.15 (10)
N2—C4—C5—O3	174.18 (9)	C6B—N3B—C10B—C9B	0.02 (16)
C3—C4—C5—O3	−4.60 (16)	C8B—C9B—C10B—N3B	−0.08 (15)
C10—N3A—C6A—N4A	−178.84 (10)	C11B—C9B—C10B—N3B	179.57 (10)
C10—N3A—C6A—C7A	1.05 (16)		

### Hydrogen-bond geometry (Å, °)

**Table:**

*D*—H···*A*	*D*—H	H···*A*	*D*···*A*	*D*—H···*A*
N2—H1N2···O4^i^	0.931 (16)	1.871 (16)	2.7912 (12)	169.7 (15)
N4A—H3NA···O1W^ii^	0.861 (18)	2.024 (17)	2.8520 (14)	161.2 (17)
N3B—H1NB···O3^iii^	0.900 (17)	1.755 (17)	2.6483 (12)	171.4 (16)
N4B—H2NB···O4^iii^	0.914 (18)	2.022 (18)	2.9323 (13)	173.8 (16)
N4B—H3NB···O3W^iv^	0.889 (18)	2.007 (18)	2.8641 (13)	161.6 (17)
N3A—H1NA···O2^iv^	0.942 (18)	1.732 (18)	2.6686 (12)	172.8 (17)
N4A—H2NA···O1^iv^	0.907 (18)	2.106 (18)	3.0021 (13)	169.4 (15)
O1W—H1W1···O3	0.871 (19)	1.902 (19)	2.7517 (12)	164.8 (17)
O1W—H2W1···O3W^iv^	0.85 (2)	1.94 (2)	2.7878 (14)	178 (2)
O2W—H1W2···O1	0.850 (18)	2.003 (18)	2.8427 (12)	169.8 (17)
O2W—H2W2···O1^v^	0.858 (18)	1.987 (18)	2.8434 (13)	176.1 (15)
O3W—H1W3···O2	0.888 (17)	1.844 (17)	2.7299 (12)	174.8 (15)
O3W—H2W3···O2W^vi^	0.881 (18)	1.900 (18)	2.7758 (13)	172.1 (17)
C10—H10A···O2W	0.93	2.50	3.3986 (15)	164

Symmetry codes: (i) −*x*+1, −*y*+1, −*z*; (ii) −*x*+2, −*y*, −*z*+1; (iii) −*x*+1, −*y*, −*z*; (iv) *x*, *y*−1, *z*; (v) −*x*+1, −*y*+1, −*z*+1; (vi) *x*, *y*+1, *z*.
